# BMI and its association with death and the initiation of renal replacement therapy (RRT) in a cohort of patients with chronic kidney disease (CKD)

**DOI:** 10.1186/s12882-019-1513-9

**Published:** 2019-08-22

**Authors:** Zaimin Wang, Jianzhen Zhang, Samuel Chan, Anne Cameron, Helen G. Healy, Sree K. Venuthurupalli, Ken-Soon Tan, Wendy E. Hoy

**Affiliations:** 1 NHMRC CKD.CRE and CKD.QLD, Health Science Building, Level 8, University of Queensland, RBWH, Brisbane, Herston, QLD 4029 Australia; 2 Centre for Chronic Disease, Health Science Building, Level 8, University of Queensland, RBWH, Brisbane, Herston, QLD 4029 Australia; 3 Kidney Health Services, Metro North Hospital and Health Service, Royal Brisbane and Women’s Hospital, Brisbane, Herston, QLD 4029 Australia; 4 Renal Services, Darling Downs Hospital and Health Service, Toowoomba Hospital, Toowoomba, QLD 4035 Australia; 5 Department of Nephrology, Logan Hospital, Metro South Hospital and Health Service, Logan, QLD 4131 Australia

**Keywords:** BMI, Obesity, Chronic kidney disease, Epidemiology, Mortality, RRT

## Abstract

**Background:**

A survival advantage associated with obesity has often been described in dialysis patients. The association of higher body mass index (BMI) with mortality and renal replacement therapy (RRT) in preterminal chronic kidney disease (CKD) patients has not been established.

**Methods:**

Subjects were patients with pre-terminal CKD who were recruited to the CKD.QLD registry. BMI at time of consent was grouped as normal (BMI 18.5–24.9 kg/m^2^), overweight (BMI 25–29.9 kg/m^2^), mild obesity (BMI 30–34.9 kg/m^2^) and moderate obesity+ (BMI ≥ 35 kg/m^2^) as defined by WHO criteria. The associations of BMI categories with mortality and starting RRT were analysed.

**Results:**

The cohort consisted of 3344 CKD patients, of whom 1777 were males (53.1%). The percentages who had normal BMI, or were overweight, mildly obese and moderately obese+ were 18.9, 29.9, 25.1 and 26.1%, respectively. Using people with normal BMI as the reference group, and after adjusting for age, socio-economic status, CKD stage, primary renal diagnoses, comorbidities including cancer, diabetes, peripheral vascular disease (PVD), chronic lung disease, coronary artery disease (CAD), and all other cardiovascular disease (CVD), the hazard ratios (HRs, 95% CI) of males for death without RRT were 0.65 (0.45–0.92, *p* = 0.016), 0.60 (0.40–0.90, *p* = 0.013), and 0.77 (0.50–1.19, *p* = 0.239) for the overweight, mildly obese and moderately obese+. With the same adjustments the hazard ratios for death without RRT in females were 0.96 (0.62–1.50, *p* = 0.864), 0.94 (0.59–1.49, *p* = 0.792) and 0.96 (0.60–1.53, *p* = 0.865) respectively.

In males, with normal BMI as the reference group, the adjusted HRs of starting RRT were 1.15 (0.71–1.86, *p* = 0.579), 0.99 (0.59–1.66, *p* = 0.970), and 0.95 (0.56–1.61, *p* = 0.858) for the overweight, mildly obese and moderately obese+ groups, respectively, and in females they were 0.88 (0.44–1.76, *p* = 0.727), 0.94 (0.47–1.88, *p* = 0.862) and 0.65 (0.33–1.29, *p* = 0.219) respectively.

**Conclusions:**

More than 80% of these CKD patients were overweight or obese. Higher BMI seemed to be a significant “protective” factor against death without RRT in males but there was not a significant relationship in females. Higher BMI was not a risk factor for predicting RRT in either male or female patients with CKD.

## Introduction

Obesity was one of the major themes of World Kidney Day in 2017 [1]. Over the past two decades, a multitude of observational studies have illustrated that obese patients on renal replacement therapy (RRT) have better survival than those with normal BMI. This concept of “reverse epidemiology” has also been observed in patients with heart failure, chronic obstructive pulmonary disease, liver cirrhosis and metastatic cancer, as well as in the geriatric population [2–7]. Recent studies have confirmed this “obesity paradox” in contemporary cohorts across different ethnicities and races, as well as different geographic regions on the world [2–7].

In a multicenter prospective observational cohort study of 2833 patients in Korea on maintenance hemodialysis, individuals with higher BMI had better survival over an average follow-up of 24.2 months [8]. Some groups have also reported that obesity in non-dialysis dependent CKD was associated with lower risk for all-cause mortality [9–13].

We recently showed that patients with chronic kidney disease (CKD) in three metropolitan public renal practices in an Australian setting had strikingly higher rates of obesity than the general population [14]. We described the cross-sectional associations of obesity with clinical characteristics [14], but we did not report the outcomes of RRT and death without RRT in the context of BMI. Here our specific objectives are to examine the association between BMI and death prior to RRT and starting RRT in this same cohort, followed for up to 5 years.

## Subjects and methods

### Paticipants and variables

This is a longitudinal cohort study. Subjects were drawn from a group of 3382 patients with CKD under renal specialty care in three major metropolitan public hospitals in Queensland, Australia. These CKD patients, who were not receiving RRT, had been enrolled, with written informed consent, in the CKD.QLD registry, which began in May 2011. However, we did not include the 38 people with BMI < 18.5, suspecting that some could have been underweight as a consequence of serious morbidities coexisting with their CKD, which might confound our interpretations of findings. Thus a total of 3344 subjects were included in the analyses.

#### Exposure variable and outcomes

The exposure variable was BMI at baseline, which was grouped as normal BMI (BMI 18.5–24.9 kg/m^2^), overweight (BMI 25–29.9 kg/m^2^), mild obesity (BMI 30–34.9 kg/m^2^) and moderate obesity+ (BMI ≥ 35 kg/m^2^), as defined by WHO criteria [15].

Outcomes were all-cause mortality without RRT and starting RRT. Participants were followed up from the date of their consent until death or starting RRT or censoring on 30 June, 2016.

#### Co-variables

Co-variables included as potential confounders in multivariate analyses, based on data availability and results of univariate analyses-based associations with outcomes, included

- Age at consent: artificially categorized as “higher” and “lower” by median values
- Gender: male and females
- CKD stages at baseline: according to the National Kidney Foundation criteria [16] ie Stage 1 if estimated glomerular filtration rate (eGFR) ≥90 ml/min; Stage 2 if eGFR≥60 & < 90; Stage 3A if eGFR≥45 & < 60; Stage 3B if eGFR≥30 & < 45; Stage 4 if eGFR≥15 & < 30 and Stage 5 if eGFR< 15.
- Primary renal diagnoses: the etiology of CKD patients was examined based on their clinical summary. Diagnoses were then categorized as glomerulonephritis (GN), genetic renal disease (GRD), diabetic nephropathy (DN), renovascular disease, others and uncertain groups.
- Major categories of comorbidities available in the cohort included: cancer (all types), diabetes, peripheral vascular disease (PVD), chronic lung disease, coronary artery disease (CAD) and all other cardiovascular disease (CVD).
- Socioeconomic status: the Socio-Economic Indexes for Areas (SEIFA) Index of Relative Socio-economic Disadvantage (IRSD) scores and deciles are produced by the Australian Bureau of Statistics to measure socio-economic status by postcodes [17]. In the analysis, IRSD scores were grouped into quintiles (i.e. highest, high, middle, low and lowest) where the highest quintile represents postcodes with the highest IRSD scores (the least disadvantaged areas) and the lowest quintile represents postcodes with the lowest IRSD scores (the most disadvantage areas).

### Statistical analyses

Crude incidence rates of mortality without RRT and starting RRT were compared across gender and BMI categories. Cox regression modelling was performed to determine hazard ratios (HRs) for mortality and starting RRT by BMI categories and other co-variables. All analyses were undertaken using Stata 14.1 (Stata Corp. Stata Statistical Software: Release 14.1, College Station. TX: StatCorp LP, 2016). Statistical significance was defined as a *p* value < 0.05 (two-tail). For those who were excluded in the analyses due to missing information on some co-variables, their profiles of age and BMI were compared with those included in the model to examine if the potential selective bias may occur.

### Ethical approval

Written informed consent to participate was obtained from patients. This study was approved by the Royal Brisbane and Women’s Hospital Ethics Committee of Queensland Health (HREC/15/QRBW/294) and the Ethics Committee of The University of Queensland (No.2011000029), Australia.

## Results

### Sample size and characteristics of cohort

Of 3344 CKD patients with baseline BMI data, 46.9% (*n* = 1567) were females and 53.1% (*n* = 1777) were males. The age of subjects at consent ranged from 18 to 100 years old with median of 68 years (67 and 68 years old for females and males, respectively). Only 7.1 and 11.9% patients were at CKD stages 1 and 2 respectively, stage 3 (A + B) accounted for 50%, while stages 4 and 5 accounted for 24.5 and 6.6%, respectively. Renovascular diseases (30.4%) and diabetic nephropathy (23.9%) were the two major primary renal diagnoses. The prevalence of major comorbidities including diabetes, PVD, cancer (all types), chronic lung disease, CAD and all other CVD were 45.9, 10.5, 22.2, 22.5, 25 and 40%, respectively. The percentages in lowest IRSD quintile (i.e the most disadvantage areas), low, middle, high and highest IRSD quintile (i.e the least disadvantage areas) were 24.7, 21.4, 22.9, 21.2 and 9.8%, respectively.

### Profiles of overweight and obesity

The proportions who were of normal BMI, overweight, mildly obese and moderately obese+ were 18.9, 29.9, 25.1 and 26.1%, respectively. The overall prevalence of overweight or obesity was 81.1% (females 79.1% vs. males 82.9%, *p* = 0.005). Mean and median BMI were 30.8 (kg/m^2^) vs. 31.4 (kg/m^2^) and 30 (kg/m^2^) vs. 30.3 (kg/m^2^) for males and females, respectively.

Table 1 shows the baseline characteristics in this cohort across different weight groups. Moderately obese+ patients had a slightly younger mean age compared with those in other BMI categories. The different weight groups had significantly different characteristics of gender, CKD stage 2 to CKD stage 4, major PRDs, comorbidities such as diabetes, cancer (all types), chronic lung disease, CAD, all other CVD and low to middle IRSD quintiles (*p* < 0.001, Table 1).

**Table 1:** Baseline characteristics

	Normal weight	Overweight	Mild obesity	Moderate obesity +		All
	(*n* = 632)	(*n* = 1001)	(*n* = 840)	(*n* = 871)	*p* value	(*n* = 3344)
Mean age, yr. (SD)	64.4 (19.1)	66.5 (15.9)	65.3 (14.8)	62.6 (13.0)	*p* < 0.001	68.0 (15.7)
Gender, *n* (%)						
Male	304 (17.1)	583 (32.8)	486 (27.4)	404 (22.7)	*P* < 0.001	1777 (100.0)
Female	328 (20.9)	418 (26.7)	354 (22.6)	467 (29.8)	*P* < 0.001	1567 (100.0)
CKD stages, *n* (%)						
Stage 1	46 (19.5)	68 (28.8)	59 (25.0)	63 (26.7)	*p* = 0.2115	236 (100.0)
Stage 2	60 (15.1)	120 (30.2)	100 (25.2)	117 (29.5)	*p* < 0.001	397 (100.0)
Stage 3A	107 (17.7)	180 (29.8)	168 (27.8)	149 (24.7)	*p* < 0.001	604 (100.0)
Stage 3B	181 (17.0)	321 (30.1)	271 (25.4)	295 (27.6)	*p* < 0.001	1068 (100.0)
Stage 4	181 (22.1)	243 (30.0)	190 (23.2)	204 (24.9)	*p* = 0.01	818 (100.0)
Stage 5	57 (25.8)	69 (31.2)	52 (23.5)	43 (19.5)	*p* = 0.094	221 (100.0)
PRD, *n* (%)						
Glomerulonephritis	82 (19.2)	140 (32.7)	110 (25.7)	96 (22.4)	*p* < 0.001	428 (100.0)
Genetic renal disease	58 (29.3)	75 (37.9)	36 (18.2)	29 (14.7)	*p* < 0.001	198 (100.0)
Diabetic nephropathy	82 (10.3)	162 (20.3)	224 (28.0)	331 (41.4)	*p* < 0.001	799 (100.0)
Renovascular disease	202 (19.9)	342 (33.7)	255 (25.1)	216 (21.3)	*p* < 0.001	1015 (100.0)
Uncertain	48 (27.6)	54 (31.0)	35 (20.1)	37 (21.2)	*p* < 0.001	174 (100.0)
Others	160 (21.9)	228 (31.2)	180 (24.7)	162 (22.2)	*p* = 0.1309	730 (100.0)
Diabetes, *n* (%)						
Yes	167 (10.9)	366 (23.9)	428 (27.9)	572 (37.3)	*p* < 0.001	1533 (100.0)
No	465 (25.7)	634 (35.1)	411 (22.7)	299 (16.5)	*p* < 0.001	1809 (100.0)
Cancer, *N* (%)						
Yes	144 (19.9)	234 (32.3)	177 (24.5)	169 (23.3)	*p* < 0.001	724 (100.0)
No	478 (18.9)	739 (29.2)	634 (25.0)	682 (26.9)	*p* < 0.001	2533 (100.0)
PVD, *n* (%)						
Yes	70 (20.5)	99 (29.0)	83 (24.3)	90 (26.3)	*p* = 0.1543	342 (100.0)
No	552 (18.9)	874 (30.0)	728 (25.0)	761 (26.1)	*p* < 0.001	2915 (100.0)
Chronic lung disease						
Yes	143 (19.1)	221 (29.5)	170 (22.7)	216 (28.8)	*p* < 0.001	750 (100.0)
No	489 (18.9)	778 (30.0)	669 (25.8)	655 (25.3)	*p* < 0.001	2591 (100.0)
CAD						
Yes	150 (17.9)	239 (28.6)	221 (26.4)	226 (27.0)	*p* < 0.001	836 (100.0)
No	482 (19.2)	762 (30.4)	619 (24.7)	645 (25.7)	*p* < 0.001	2508 (100.0)
All other CVD						
Yes	247 (18.5)	389 (29.1)	319 (23.8)	383 (28.6)	*p* < 0.001	1338 (100.0)
No	385 (19.2)	612 (30.5)	521 (26.0)	488 (24.3)	*p* < 0.001	2006 (100.0)
IRSD quintiles, *n* (%)						
Lowest	109 (13.9)	230 (29.3)	205 (26.1)	242 (30.8)	*p* < 0.001	786 (100.0)
Low	116 (17.1)	215 (31.6)	163 (24.0)	186 (27.4)	*p* < 0.001	680 (100.0)
Middle	144 (19.8)	209 (28.7)	211 (29.0)	164 (22.5)	*p* < 0.001	728 (100.0)
High	152 (22.6)	197 (29.3)	156 (23.2)	168 (25.0)	*p* = 0.061	673 (100.0)
Highest	77 (24.8)	97 (31.2)	72 (23.2)	65 (20.9)	*p* = 0.063	311 (100.0)

Notes: Normal weight: BMI 18.5–24.9 kg/m^2^; Overweight: BMI 25.0–29.9; Obesity: 30–34.9; Moderate obesity +: BMI > =35. *P*-value: ANOVA test for continuous variables and Goodness-of-fit tests for categorical variables on multinomial data

### Association of overweight or obesity with mortality and starting RRT

The outcomes of this CKD cohort were followed up until June 2016. The total follow up was 12,087 person years for the 3344 patients, with individual ranges from 0 to 5.5 years. The mean and median follow up years were 3.6 and 3.9 years, respectively. A total of 413 people died without RRT (234 males and 179 females), and 219 patients started RRT (143 males and 76 females). Among those who died without RRT, primary cause of death was reported as cardiovascular events in 61%, cancer (all types) in 17.1%, and end stage kidney failure (ESKF) in 22%.

**Fig. 1:**
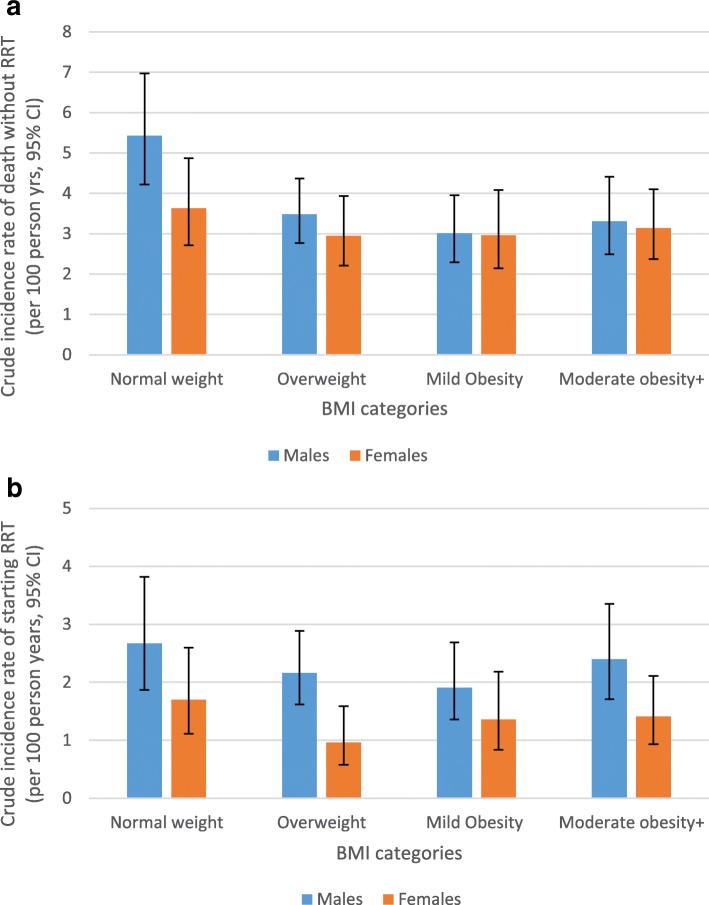
**a** Crude incidence rate of death without RRT (per 100 person years, 95% CI) by BMI categories and gender. **b** Crude incidence rate of starting RRT (per 100 person years, 95% CI) by BMI categories and gender

There was no significant difference in the crude incidence rate of death without RRT (per 100 person years) between males and females (rate, 95% CI: 3.66 (3.22–4.16) vs. 3.15 (2.72–3.64), *p* = 0.246). However the crude incidence rate of starting RRT was significantly higher in males than females (2.34 (1.90–2.64) vs.1.34 (1.07–1.67), *p* = 0.001). As shown in Fig. 1a, the crude incidence rate of death without RRT was significantly higher in male patients who were normal weight, (5.43 (4.22–6.97)) compared to those who were overweight (3.48 (2.77–4.37), *p* = 0.022), mildly obese (3.01 (2.29–3.95), *p* = 0.007) and moderately obese+ (3.31 (2.49–4.41), *p* = 0.033). There were similar trends in females but the differences were not statistically significant (Fig. 1a). Analyses also show that the primary cause of CVD, cancer (all types) and ESKF death was not significantly different among BMI categories (data not included). Figure 1b shows that the crude incidence rate of starting RRT per 100 person years was higher in patients with normal weight compared to those who were overweight, mildly obese and moderately obese+ in both males and females, but the differences were not statistically significant (Fig. 1b).

**Fig. 2:**
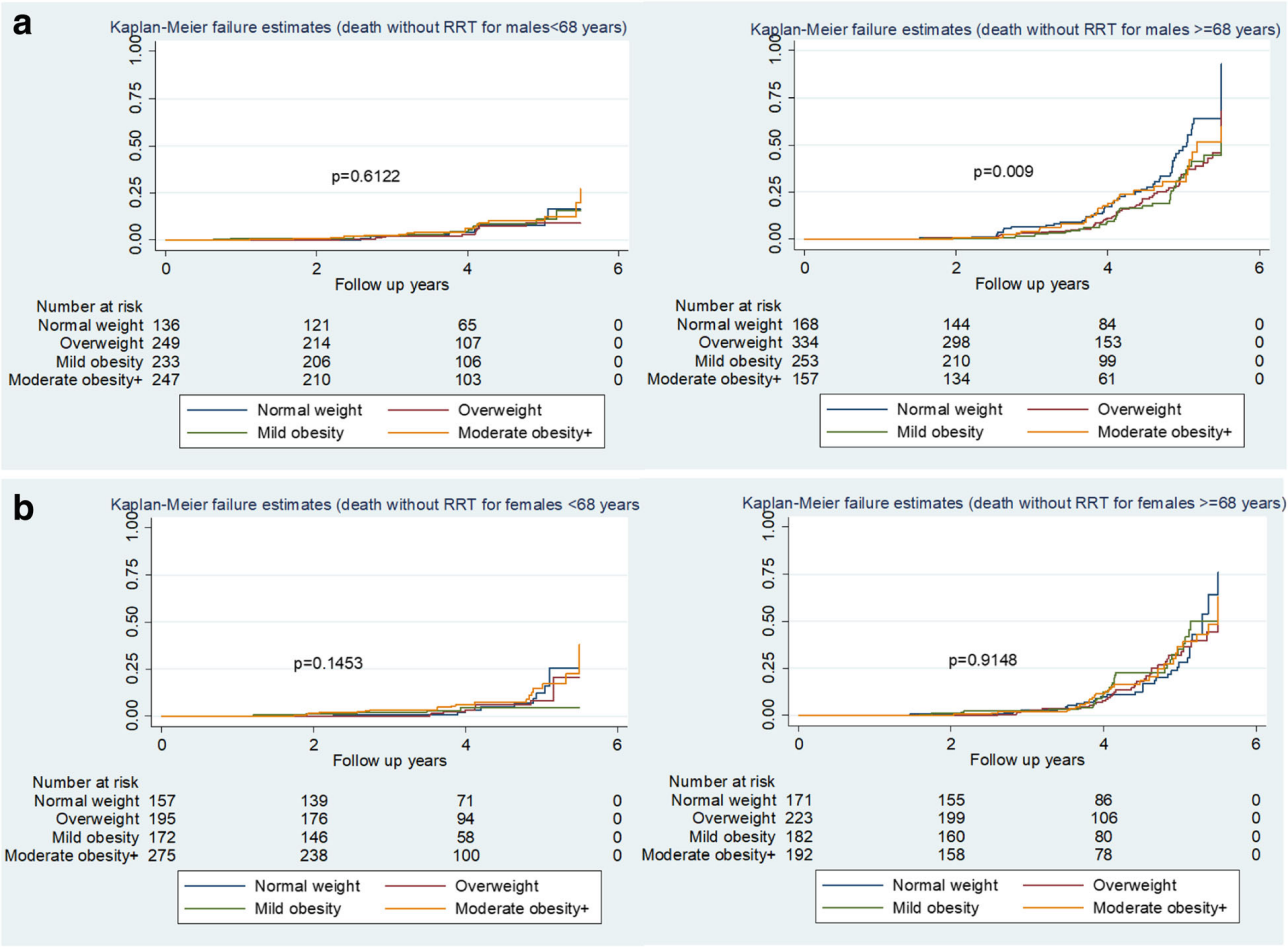
**a** Kaplan_Meier estimates for death without RRT by BMI categories (Males). **b** Kaplan_Meier estimates for death without RRT by BMI categories (Females)

**Fig. 3:**
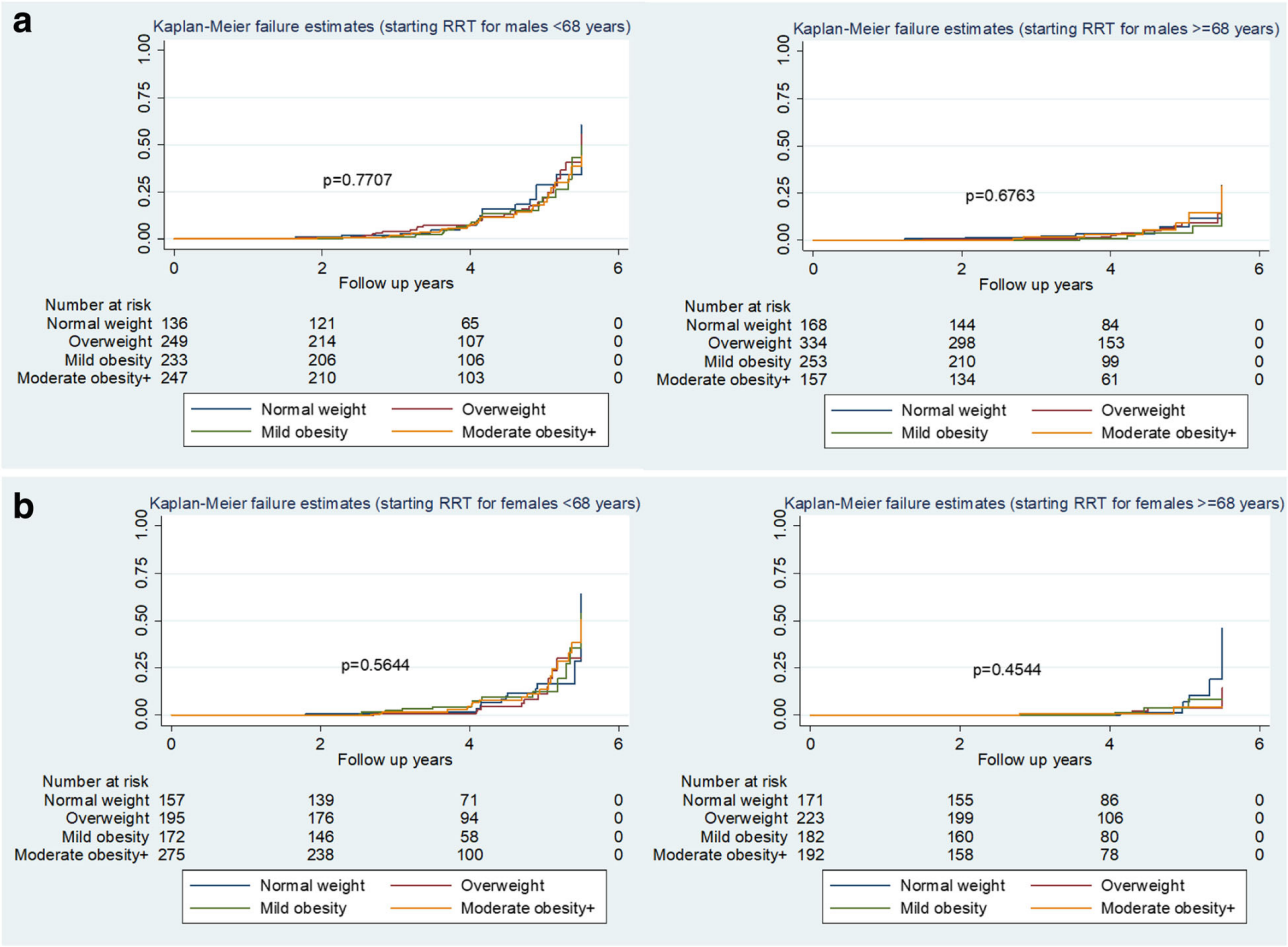
**a** Kaplan Meier estimates for starting RRT by BMI categories (Males). **b** Kaplan Meier estimates for starting RRT by BMI categories (Females)

Kaplan-Meier survival curves with associated risk tables (Fig. 2a and b) show the higher rates of death without RRT in older subjects (here expressed as ≥ 68 years of age at consent, the group median age), and amongst older males, with a significant difference by BMI categories (log rank test *P* = 0.009) (Fig. 2a). The probability estimates of Kaplan-Meier survival curves for death without RRT at 5- year follow up were 0.51 for normal weight and 0.66 for both overweight and mildly obese males, and 0.70 for moderately obese+ males aged ≥ 68 years. There were no significant differences in female survivals by BMI category (Fig. 2b). Kaplan-Meier survival curves for RRT show higher rates for younger than older people, and no significant differences by BMI category in males or females (Fig. 3a, b).

Of 3344 CKD patients with baseline BMI data, 50 males (2.8% out of 1777 males) and 37 females (2.4% out of 1567 females) were excluded in the multivariate models due to the missing data for some co-variables. As the means of age and BMI were not significantly different between those missing patients and those patients in the models, the bias due to missing data in this study were ignored; thus a total of 3257 CKD patients were included in the final models. Those co-variables that were significantly associated with the outcomes in the univariate analysis were included in the final multivariate models.

Using people of normal BMI as the reference group, and after adjusting for age, CKD stage at consent, primary renal diagnoses, comorbidities of cancer, diabetes, PVD, chronic lung disease, CAD and all other CVD, and for IRSD quintiles, the hazard ratios (HRs, 95% CI) for death without RRT in males were 0.65 (0.45–0.92, *p* = 0.016), 0.60 (0.40–0.90, *p* = 0.013), and 0.77 (0.50–1.19, *p* = 0.239) for the overweight, mildly obese and moderately obese+ respectively (Table 2), and for females were 0.96 (0.62–1.50, *p* = 0.864), 0.94 (0.59–1.49, *p* = 0.792) and 0.96 (0.60–1.53, *p* = 0.865) respectively (Table 3). In males, the adjusted HRs for starting RRT were 1.15 (0.71–1.86, *p* = 0.579), 0.99 (0.59–1.66, *p* = 0.970), and 0.95 (0.56–1.61, *p* = 0.858) for the overweight, mildly obese and moderately obese+ groups, respectively (Table 4), and in females they were 0.88 (0.44–1.76, *p* = 0.727), 0.94 (0.47–1.88, *p* = 0.862) and 0.65 (0.33–1.29, *p* = 0.219) respectively (Table 5).

**Table 2:** Prediction of mortality without RRT (Males)

Variables	HRs	95% CI		*p*
BMI categories				
Normal BMI	Reference group			
Overweight	0.65	0.45	0.92	0.016
Mild obesity	0.60	0.40	0.90	0.013
Moderate obesity+	0.77	0.50	1.19	0.239
Age > =68 years	2.33	1.64	3.32	< 0.001
CKD stage				
Stage 1	Reference group			
Stage 2	2.17	0.26	17.90	0.471
Stage 3A	3.22	0.43	24.39	0.257
Stage 3B	3.22	0.44	23.77	0.252
Stage 4	5.43	0.74	39.88	0.096
Stage 5	3.96	0.53	29.63	0.181
Primary renal diagnoses				
Glomerulonephritis	Reference group			
Genetic renal disease	2.65	0.74	9.53	0.136
Diabetic nephropathy	4.15	1.72	9.99	0.002
Renovascular disease	3.90	1.67	9.10	0.002
Cancer	1.34	1.01	1.78	0.041
Diabetes	1.21	0.85	1.70	0.288
PVD	1.48	1.07	2.04	0.017
Chronic lung disease	1.14	0.86	1.52	0.374
CAD	1.78	1.32	2.40	< 0.001
All other CVD	1.24	0.89	1.71	0.203
IRSD quintiles				
Lowest	Reference group			
Low	0.93	0.63	1.37	0.718
Middle	0.60	0.40	0.90	0.014
High	0.60	0.40	0.91	0.015
Highest	0.52	0.32	0.86	0.011

**Table 3:** Prediction of mortality without RRT (Females)

Variables	HRs	95% CI		*p*
BMI categories				
Normal BMI	Reference group			
Overweight	0.96	0.62	1.50	0.864
Mild obesity	0.94	0.59	1.49	0.792
Moderate obesity+	0.96	0.60	1.53	0.865
Age > =68 years	2.00	1.37	2.93	< 0.001
CKD stage				
Stage 1	Reference group			
Stage 2	3.89	0.48	31.63	0.204
Stage 3A	3.85	0.48	30.86	0.205
Stage 3B	5.10	0.68	38.17	0.113
Stage 4	8.45	1.14	62.72	0.037
Stage 5	9.93	1.31	75.45	0.027
Primary renal diagnoses				
Glomerulonephritis	Reference group			
Genetic renal disease	0.35	0.08	1.59	0.175
Diabetic nephropathy	0.90	0.43	1.86	0.769
Renovascular disease	0.67	0.34	1.34	0.263
Cancer	1.76	1.25	2.46	0.001
Diabetes	1.53	1.02	2.29	0.042
PVD	1.46	0.94	2.26	0.093
Chronic lung disease	1.35	0.95	1.92	0.096
CAD	1.40	0.98	2.00	0.068
All other CVD	1.54	1.09	2.18	0.015
IRSD quintiles				
Lowest	Reference group			
Low	0.86	0.52	1.40	0.534
Middle	0.81	0.52	1.25	0.338
High	0.88	0.56	1.37	0.572
Highest	0.76	0.42	1.37	0.369

**Table 4:** Prediction of starting RRT (Males)

Variables	HRs	95% CI		*p*
BMI categories				
Normal BMI	Reference group			
Overweight	1.15	0.71	1.86	0.579
Mild obesity	0.99	0.59	1.66	0.970
Moderate obesity+	0.95	0.56	1.61	0.858
Age > =68 years	0.26	0.17	0.39	< 0.001
CKD stage				
Stage 1 + 2	Reference group			
Stage 3A + 3B	7.95	1.05	60.26	0.045
Stage 4 + 5	81.76	11.32	590.52	< 0.001
Primary renal diagnoses				
Glomerulonephritis	Reference group			
Genetic renal disease	1.52	0.69	3.34	0.299
Diabetic nephropathy	1.89	0.94	3.84	0.076
Renovascular disease	1.27	0.65	2.47	0.478
Cancer	0.73	0.43	1.23	0.236
Diabetes	0.95	0.56	1.61	0.842
PVD	1.09	0.67	1.76	0.737
Chronic lung disease	0.92	0.58	1.45	0.721
CAD	0.67	0.43	1.03	0.069
All other CVD	0.98	0.65	1.47	0.913
IRSD quintiles				
Lowest	Reference group			
Low	1.02	0.61	1.71	0.949
Middle	0.82	0.51	1.33	0.419
High	0.88	0.50	1.53	0.648
Highest	0.77	0.42	1.42	0.403

**Table 5:** Prediction of starting RRT (Females)

Variables	HRs	95% CI		*p*
BMI categories				
Normal BMI	Reference group			
Overweight	0.88	0.44	1.76	0.727
Mild obesity	0.94	0.47	1.88	0.862
Moderate obesity+	0.65	0.33	1.29	0.219
Age > =68 years	0.19	0.10	0.37	< 0.001
CKD stage				
Stage 1 + 2	Reference group			
Stage 3A + 3B	3.35	0.37	30.42	0.283
Stage 4 + 5	62.27	8.44	459.66	< 0.001
Primary renal diagnoses				
Glomerulonephritis	Reference group			
Genetic renal disease	1.28	0.48	3.37	0.622
Diabetic nephropathy	2.47	0.86	7.09	0.092
Renovascular disease	0.96	0.36	2.59	0.938
Cancer	0.98	0.47	2.04	0.953
Diabetes	0.52	0.24	1.14	0.102
PVD	0.81	0.33	1.95	0.635
Chronic lung disease	1.30	0.70	2.40	0.412
CAD	0.65	0.29	1.43	0.280
All other CVD	0.96	0.53	1.73	0.890
IRSD quintiles				
Lowest	Reference group			
Low	0.87	0.40	1.90	0.733
Middle	0.72	0.36	1.44	0.351
High	1.01	0.50	2.05	0.969
Highest	1.27	0.48	3.41	0.630

## Discussion

A larger proportion of the non-dialysis CKD population in this Australian study is overweight or obese. Fifty one point two (51.2%) percent of subjects were obese, compared with 28.4% of a contemporaneous National Health Survey (NHS) sample of the general Australian population [18]. Even patients without diabetes in our CKD study population were notably more often overweight or obese than the NHS sample population [14, 18]. Although obesity is widely considered a health risk [19], when followed for 5 years, overweight or mildly obese patients in this cohort of patients with CKD, particularly those males who were above the group median age of 68 years at enrolment, had a significantly lower risk for death without RRT than males with a normal BMI. There were no significant associations between higher BMI and starting RRT in either males or females.

The phenomenon of lower mortality in older males with CKD who were overweight or obese is consistent with recent studies in which dialysis patients of higher BMI had lower rates of death than those of “normal” BMI, termed the “obesity paradox” [8, 20]. The explanation for this phenomenon remains elusive. Does it represent exacerbated mortality risk in “normal” BMI patients and “protective” factors in overweight and obese subjects, or contributions from both [21, 22]? The phenomenon persists with adjustment for age, so that lower BMIs in older patients are not the major explanation.

There were no clear differences in “cause” of death by BMI category, with about 60% in each BMI category dying with cardiovascular conditions, comparable percentages dying with cancer (about 16%) and marginally fewer people of normal BMI (17.0%) dying with ESKF than those of higher BMIs (23%). The association persisted after adjustment for all the common co-morbidities that were independently associated with mortality risk.

Some have speculated that overweight or obese CKD patients may have better and more frequent care than others, and have adopted a healthier lifestyle. However, the standard settings and policies in these major public specialty CKD practices make it unlikely that normal weight CKD patients are relatively neglected in these domains of health care. Similarly, better nutritional status in those CKD patients with higher BMI may conceivably be a protective factor for mortality. However, no data of nutritional status were collected in this cohort. Others propose that CKD patients of higher BMI may better tolerate the side effects of some interventions [23, 24]. We note, too, that the patients’ BMIs in our report were ascertained more than 3.5 years prior to death on average, and might have changed substantially since first measured. Weight loss is common with progression of kidney diseases and BMIs close to death might have been substantially lower than documented on recruitment into the CKD.QLD cohort [23]. It has also been argued that, because CKD patients are more likely to develop fluid retention, BMI might not be an ideal measure for examining the risk in CKD [23], and using both BMI and waist circumference (WC), which reflects visceral adiposity, would better represent the associations between muscle mass, adipose tissue and various causes of death in CKD [21, 25–29]. Some of these factors seem unlikely to be major contributors to this phenomenon. There are undoubtedly also unrevealed confounders affecting this association. Given that this “reverse epidemiology” applies in many settings, groups of patients and conditions, CKD-specific explanations can have only limited applicability. An intuitive explanation might be that, in a population where most adults are overweight or obese, the presence of lower or “normal BMIs” can flag significant health disadvantage or frank illness for a substantial proportion of people. Our exclusion of patients with BMIs < 18.5 in this analysis would not entirely mitigate against this influence.

Major strengths of this study include the large cohort of well-studied CKD patients, followed up for 5 years, the availability of data on all-cause mortality without RRT and on starting RRT, and ability to adjust for some important comorbidities including cancer, diabetes, PVD, chronic lung disease, CAD and all other CVD, which are potentially related to the risk of mortality. Their continued follow up will expose more findings over time, as numbers of death and RRT starts increase. However, it should be emphasised that these CKD patients do not represent the general community-based CKD population, as this cohort is selected for severity by being already referred to renal specialty practices. In addition some other confounders that may affect the associations were not included in our analyses.

## Conclusions

In summary, more than 80% of these CKD patients were overweight or obese. Higher BMI seemed to be a significant “protective” factor against death without RRT in males, but there was not a significant relationship in females. Higher BMI was not a risk factor for predicting RRT in either males or female CKD patients.

## Data Availability

The datasets used and/or analysed during the current study are available from the corresponding author on reasonable request.

## Abbreviations

CAD: Coronary artery disease
CKD: Chronic kidney disease
eGFR: Estimated glomerular filtration rate
ESKF: End stage kidney failure
IRSD: Index of Relative Socio-Economic Disadvantage
NHS: National Health Survey
PVD: Peripheral vascular disease
RRT: Renal replacement therapy
SEIFA: Socio-Economic Indexes for Areas
WC: Waist circumference
